# Antibacterial Activity of New Oxazolidin-2-One Analogues in Methicillin-Resistant *Staphylococcus aureus* Strains

**DOI:** 10.3390/ijms15045277

**Published:** 2014-03-26

**Authors:** Jesús Córdova-Guerrero, Esteban Hernández-Guevara, Sandy Ramírez-Zatarain, Marco Núñez-Bautista, Adrián Ochoa-Terán, Raquel Muñiz-Salazar, Julio Montes-Ávila, Gabriela López-Angulo, Armando Paniagua-Michel, Gustavo A. Nuño Torres

**Affiliations:** 1 Escuela de Ciencias de la Salud, Universidad Autónoma de Baja California, Ensenada, Baja California 22890, México; E-Mails: jesus.cordova@uabc.edu.mx (J.C.-G.); bautistamk8814@hotmail.com (M.N.-B.); arpaniagua@hotmail.com (A.P.-M.); gus_aryam19@hotmail.com (G.A.N.T.); 2 Centro de Graduados e Investigación en Química, Instituto Tecnológico de Tijuana, Tijuana, Baja California 22000, México; E-Mails: ehgm10@hotmail.com (E.H.-G.); Sandy_denys@hotmail.com (S.R.-Z.); 3 Facultad de Ciencias Químico Biológicas, Universidad Autónoma de Sinaloa, Culiacán, Sinaloa 80010, México; E-Mails: julmontes@gmail.com (J.M.-Á.); gabylpz06@gmail.com (G.L.-A.)

**Keywords:** *Staphylococcusaureus*, methicillin, oxazolidin-2-ones, antibacterial

## Abstract

*Staphylococcus aureus* is one of the most common causes of nosocomial infections. The purpose of this study was the synthesis and *in vitro* evaluation of antimicrobial activity of 10 new 3-oxazolidin-2-one analogues on 12 methicillin resistant *S. aureus* (MRSA) clinical isolates. *S. aureus* confirmation was achieved via catalase and coagulase test. Molecular characterization of MRSA was performed by amplification of the *mecA* gene. Antimicrobial susceptibility was evaluated via the Kirby-Bauer disc diffusion susceptibility test protocol, using commonly applied antibiotics and the oxazolidinone analogues. Only (*R*)-5-((*S*)-1-dibenzylaminoethyl)-1,3-oxazolidin-2-one (**7a**) exhibited antibacterial activity at 6.6 μg. These results, allow us to infer that molecules such as **7a** can be potentially used to treat infections caused by MRSA strains.

## 1. Introduction

**Figure 1.**
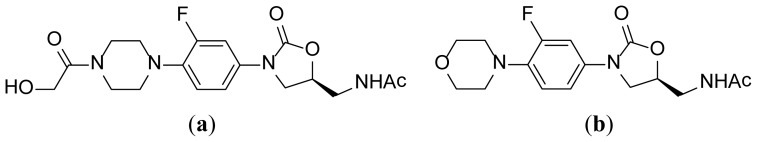
(**a**) Eperezolid and (**b**) Linezolid.

*Staphylococcus aureus* is one of the most common causes of nosocomial infections, which can occur via the direct contamination of an open wound, such as postsurgical wound infection diseases. Since the emergence of *S. aureus* isolates with resistance to methicillin [1], it has become a well-known etiological agent for a wide variety of infections. Methicillin-resistant *S. aureus* (MRSA) infections have become a common problem in hospital and community-acquired infections, and have been associated with prolonged hospital stay and increased costs. Already multi-resistant to different classes of antibiotics, MRSA had been reported to acquire resistance to gentamicin and related aminoglycosides [2]. Therefore, the treatment of infections due to these organisms and their eradication is very difficult. Methicillin resistance is mediated by the expression of the *mecA* gene, which encodes for the modified penicillin-binding protein (PBP2a or PBP2′), which has low affinity for β-lactam antibiotics and facilitates cell-wall synthesis in the presence of methicillin and other β-lactams. This gene is contained on a genetic island called the Staphylococcal chromosomal cassette mec (SCCmec). The *mecA* gene confers resistance to β-lactam derivatives, chloramphenicol, tetracyclines, macrolides, lincosamides, aminoglycosides, and even quinolones [3,4]. The commercially available drugs such as Linezolid and Eperezolid (see Figure 1) bear a 1,3-oxazolidin-2-one chemical skeleton, which have exhibited antibacterial activity against Gram-positive bacteria. Oxazolidinones represent a new class of antibacterial agents acting via a novel mechanism of action by early inhibition of protein synthesis; the 50S subunit binds to the 23S ribosomal RNA binding site, blocking the formation of the 70S functional complex, an essential component in its synthetic process [5,6]. Therefore, it is extremely important to generate new antibacterial agents against microorganisms that have undergone genetic mutation and that have shown common antibiotic resistance [7].

As a consequence, extensive efforts have been directed towards the development of novel analogs showing potent antimicrobial activity on drug resistant isolates. Wang *et al.* [8] have recently reported the synthesis and antimicrobial evaluation of chiral 1,3-oxazolidinones against *S. aureus* ATCC 29213 and ATCC 43300 as control isolates, showing a minimum inhibition concentration (MIC) value in the range of 7.4–119 μg/mL.

**Figure 2.**
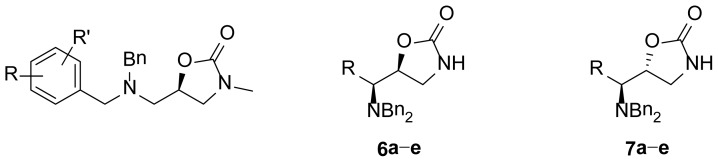
Chemical structure of chiral 5-substituted 1,3-oxazolidin-2-ones.

The purpose of this study was to synthesize a series of diasteromeric 1,3-oxazolidin-2-ones derived from α-dibenzylaminoaldehydes and evaluate the *in vitro* anti-microbial activity of ten novel chiral 1,3-oxazolidin-2-ones against MRSA isolates (see Figure 2). As can be seen, these compounds have a dibenzylamino group attached to the 1,3-oxazolidinone ring through a one carbon bridge and have an asymmetric center at the C5 position of the 1,3-oxazolidinone as in the compounds previously reported. These similitudes encourage us to evaluate the antimicrobial activity of the new chiral 1,3-oxazolidin-2-ones.

## 2. Results and Discussion

### 2.1. Chemical Synthesis

**Scheme 1.**
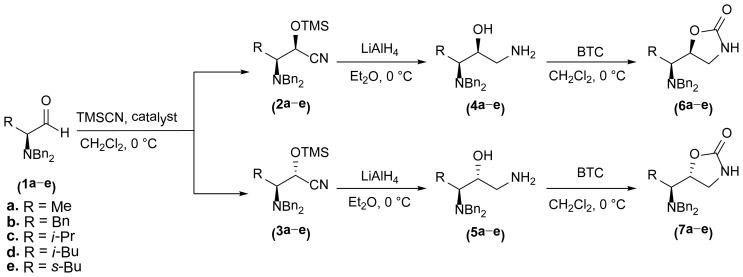
Stereoselective synthesis of compounds **6** and **7**.

The 1,3-oxazolidin-2-one analogues **6a**–**e** and **7a**–**e** were synthesized via initial reduction of α-aminoaldehydes **1a**–**e** (obtained from l-alanine, l-phenylalanine, l-isoleucine, l-leucine, and l-valine) in the presence of trimethylsilyl cyanide (TMSCN) and magnesium bromide (MgBr_2_) as the catalyst of choice to afford the silyl ethers **2a**–**e** as the predominant products, whereas in the case of the synthesis of the anti silylethers **3a**–**e**, zinc iodide (ZnI_2_) was used as the catalyst of choice, as reported by Ochoa-Terán and Rivero [9] (see Scheme 1).

Treatment of these analogues with lithium aluminum hydride (LiAlH_4_) in anhydrous diethyl ether (Et_2_O) at 0 °C allowed for the formation of the corresponding chiral amino alcohols **4** and **5**, which were treated with bis-(trichloromethyl) carbonate (BTC) to produce the desired 5-substituted-1,3-oxazolidin-2-ones in 47% to 99% yield [9] (see Table 1).

As show in Scheme 1, the stereoselectivity of products **6** and **7** was achieved during the addition of TMSCN to the chiral dibenzylaminoaldehyde **1**, giving rise to the observed stereochemical outcome (syn- and anti-addition products). It is also worth to mention that the configuration of the stereogenic center from the α-aminoaldehyde **1** is not altered along the synthetic process, whereas the configuration of the second chiral center can be manipulated in a controlled manner. The stereochemical control in this synthetic process plays a very important role in the antimicrobial evaluation of the compounds that were synthesized, since we were able to synthesize pairs of diasteroisomers in such a way that we could investigate the importance of the stereochemistry in the antibacterial activity.

### 2.2. Antimicrobial Susceptibility Analysis

Clinical samples were provided by the Instituto Mexicano del Seguro Social (IMSS) No.8 hospital, a government healthcare institution located in the city of Ensenada, Baja California, Mexico. We analyzed 12 clinical isolates identified as MRSA by biochemical tests (catalase and coagulase) and through the molecular identification of the *mecA* gene. All 12 isolates amplified the *mecA* gene showing the same haplotype, which corresponds to the reference sequence of *S. aureus* ATCC 43300 (Gene Bank Accession No. JN801172). All isolates were resistant to one, two or three antibiotics, with the exception of Vancomycin and Linezolid. Most of the isolates were resistant to Ceftazidime/Clavulanic acid (C/CA), except SMC-27 and SMC-71 isolates (see Table 2).

Among the ten oxazolidinone analogues that were tested, only [(*R*)-5-((*S*)-1-(dibenzylaminoethyl)-1,3-oxazolidin-2-one] (**7a**) showed antimicrobial activity against MRSA isolates at 6.6 μg. Only two isolates were not active to 1,3-oxazolidinone **7a**, SMC-71 that was resistant to I, and SMC-79, which was resistant to C and C/CA. In addition, we have tested some strains (SMC-27, SMC-47 and SMC-79) using the microdilution method, which was also utilized by Wang *et al.* [8]. Our results showed that the MIC of the SMC-27 and SMC-47 strains was 12.5 μg/mL for each one, a value that can be compared to the most active chiral 1,3-oxazolidinones synthesized by Wang *et al.* with MIC values in the range of 7.4–119 μg/mL. Furthermore, the SMC-79 showed no inhibition at any concentration. For the three tested strains we obtained similar results to the ones observed by using the Kirby Bauer’s disc diffusion method. As an additional experiment, the content of one of the wells where no growth was observed was cultured on an agar plate and incubated for 24 h at 36 °C, and no bacterial growth was observed, which suggests that the tested analogue **7a** presents bactericidal activity. Since we only analyzed the *mecA* gene, we are not able to determine the genetic features of the isolates SMC-71 and SMC-79 that conferred resistance to this compound. As a result, it is important to determine the type of *SCCmec* gene and the presence of *Panton-Valentine leukocidin* (PVL) toxin gene, which is associated with increased virulence of certain strains *S. aureus* [3,10–12].

### 2.3. Toxicity Test

A preliminary toxicity study was performed on the 1,3-oxazolidin-2-one analogues via a bioassay on brine shrimp. Despite the lack of specificity, this bioassay is considered a general test that indicates the possibility to detect toxicity on a certain compound. If a crude extract exhibits a LC_50_ value within the range of 1000 to 2000 parts per million (ppm) and a pure compound presents a value below 200 ppm, it is highly possible to detect toxicity when a specific bioassay is performed. Hence, the LC_50_ value that was obtained for the oxazolidinone analogue **7a** was 590 ppm. Therefore, it is possible that this analogue which exhibited antimicrobial activity could be a potential antibacterial agent with low toxicity, for the treatment of infections caused by MRSA isolates.

## 3. Experimental Section

### 3.1. Biochemical and Molecular Identification of Methicillin Resistant S. aureus (MRSA) Isolates

The clinical samples were collected in the period of August 2010 through May 2011. The samples were cultured on 5% sheep blood agar, incubated at 36 ± 1 °C for 24 to 48 h. Afterwards, a colony showing typical phenotypic features of *S. aureus* was chosen, followed by isolation on mannitol salt agar, and the coagulase and catalase tests were performed. *S. aureus* ATCC 25923 and ATCC 43300 was used as control isolates. Molecular identification of MRSA isolates was performed by polymerase chain reaction (PCR) amplification of the *mecA* gene according to François *et al.* [13]. Total genomic DNA was isolated from one bacterial colony, which was suspended in 100 μL of lysis solution [10 mM Tris-HCl (pH 8.3), 2mM MgCl_2_, 50 Mm KCl] and incubated at 95 °C for 15 min. The homogenate was centrifuged at 5000 rpm for 5 min to separate phases [13]. The supernatant was recovered and stored at −20 °C until further analysis. The quantity and quality of the genomic DNA was evaluated by agarose electrophoresis and Gelstar (Lonza Rockland, Inc., Rockland, MA, USA) stain. After centrifugation at 5000 rpm for 2 min, the supernatant was directly used in the PCR amplification assay. In order to verify the amplified fragment, the sequence was analysed. Sequence data were examined for signal quality and aligned using Codon Code Aligner version 3.0.2 (CodonCode Corporation, Centerville, TX, USA) [14]. Multiple alignments were conducted in CLUSTALX, as implemented in the software Molecular Evolutionary Genetics Analysis (MEGA version 5) (The Biodesign Institute, Tempe, AZ, USA) [15].

### 3.2. Antimicrobial Susceptibility Analysis

Antimicrobial susceptibility testing was performed using Kirby Bauer’s disc diffusion method according to performance standards of Clinical and Laboratory Standards Institute (CLSI) [16] *S. aureus* ATCC 25923 and ATCC 43300 were used as control. The panel of antibiotics tested included those that are recommended by CLSI are commonly used locally in empirical treatment of *S. aureus* infections: Ceftazidime, Norfloxacin, Imipenem, Ceftazidime/Clavulanic acid, Vancomycin, and Linezolid, as well as the ten oxazolidinone analogues **6** and **7**. Plates were incubated for 24 h at 36 °C and examined by visual observation. The discs were tested in triplicate, including one with a solvent blank and three for the standard antibiotics. Inhibitory activity was measured (mm) as the diameter of the observed inhibition zones.

### 3.3. Toxicity Test

In order to evaluate the toxicity of the oxazolidinones, a test of lethality to *Artemia salina* brine shrimp was made [17]. Briefly, dried brine shrimp eggs were bred in saline medium (Instant Ocean^®^, Blacksburg, VA, USA). After 48 h a few shrimps hatched and were ready for testing. One-day-old larvae were transferred into 96 well plates (10 per wall) containing the oxazolidinone and saline solution. The oxazolidinones were tested at 1000, 500, 200, 100, 50 and 25 ppm. In each case, three replicates of each concentration were assayed. Potassium dichromate (5 mg/mL) and 1% dimethyl sulfoxide (DMSO) in seawater were used as positive and negative controls respectively. After 24 h, the numbers of survivors were counted and percentage of death calculated. The lethal concentration fifty (LC_50_), 95% confidence interval and slope were determined from the 24 h counts using the probity analysis method described by Finney [18].

### 3.4. Chemical Synthesis

**Scheme 2.**
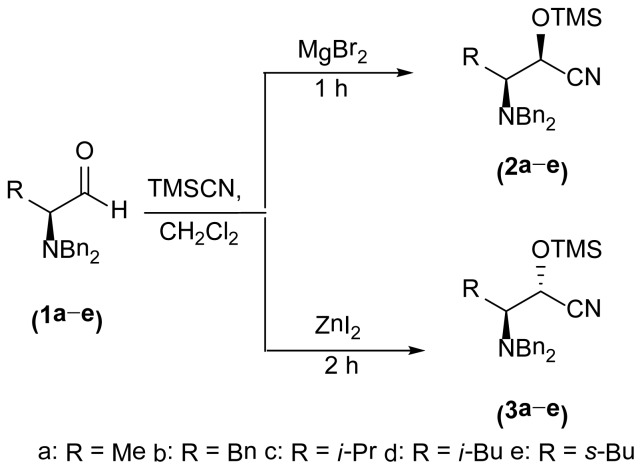
Stereoselective synthesis of silyl ethers **2** and **3**.

A general procedure for the synthesis of α-*N*,*N*-dibenzylaminotrimethyl-silyloxycyanohydrines **2** and **3** is described below (see Scheme 2).

A solution of (*S*)-2-(*N*,*N*-dibenzylamino)propanal **1** (17.3 mmol, 1 equiv) dissolved in 100 mL of anhydrous dichloromethane (DCM) was added to a 250 mL round bottom flask under nitrogen atmosphere, and ZnI_2_ (5.52 g, 17.3 mmol, 1 equiv) was added dropwise with stirring for 10 min. TMSCN (2.54 mL, 1.88 g, 19 mmol, 1.1 equiv) was added dropwise, and the resulting mixture was stirred for 2 h at 0 °C. Water (20 mL) was added and the mixture was stirred for 5 min and it was allowed to warm to 25 °C. The resulting mixture was extracted with DCM (50 mL) and the layers were separated. The aqueous layer was extracted with DCM (2 × 50 mL). The organic layers were combined and washed with brine (2 × 50 mL), dried over anhydrous sodium sulfate (Na_2_SO_4_), filtered, and the solvent was removed under vacuum to render the corresponding compound **3** as a colorless oil. In the case of the synthesis of silyloxycyanohydrines **2**, MgBr_2_ (17.3 mmol, 1 equiv) was used as the catalyst of choice to obtain the desired product **2** as colorless oil.

(2*S*,3*S*)-3-(Dibenzylamino)-2-(trimethylsilyloxy) butanonitrile (**2a**). Colorless oil. (5.5 g, 13.8 mmol, 88%). IR (neat, cm^−1^): 3061, 2955, 2228, 1255, 1106, 848 cm^−1. 1^H NMR (200 MHz, CDCl_3_) δ 7.34 (10H, m), 4.45 (1H, d, *J* = 5.8 Hz), 3.81 (2H, d, *J* = 13.8 Hz), 3.63 (2H, d, *J* = 13.8 Hz), 3.00 (1H, m), 1.75 (2H, m), 1.25 (1H, m), 0.90 (3H, d, *J* = 6.6 Hz), 0.70 (3H, d, *J* = 6.6 Hz). ^13^C NMR (50 MHz, CDCl_3_) δ 139.0, 128.6, 128.0, 126.8, 119.8, 63.2, 58.8, 54.6, 36.4, 25.0, 23.3, 22.0, 20.4.

(2*R*,3*S*)-3-(Dibenzylamino)-4-phenyl-2-(trimethylsilyloxy) butanonitrile (**2b**). Colorless oil. (3.8 g, 8.9 mmol, 95%). IR (neat, cm^−1^): 3029, 2951, 2367, 1255, 1113, 849 cm^−1. 1^H NMR (200 MHz, CDCl_3_) δ 7.28 (15H, m), 4.48 (1H, d, *J* = 6.2 Hz), 3.72 (4H, br s), 3.38 (1H, dt, *J* = 8.0, 6.2 Hz), 3.04 (2H, m), 1.26 (9H, s). ^13^C NMR (50 MHz, CDCl_3_) δ 139.3, 138.7, 129.4, 128.8, 127.5, 127.0, 126.8, 126.3, 119.7, 64.2, 62.8, 55.2, 33.2, 1.9.

(2*S*,3*S*)-3-(Dibenzylamino)-4-methyl-2-(trimethylsilyloxy) hexanonitrile (**2c**). Colorless oil. (3.9 g, 9.9 mmol, 99%). IR (neat, cm^−1^): 3061, 2959, 2233, 1246, 1103, 846 cm^−1. 1^H NMR (200 MHz, CDCl_3_) δ 7.33 (10H, m), 4.78 (1H, d, *J* = 4.8 Hz), 3.87 (2H, d, *J* = 13.6 Hz), 3.65 (2H, d, *J* = 13.6 Hz), 2.79 (1H, dd, *J* = 7.8, 4.8 Hz), 1.94 (2H, m), 1.17 (1H, m), 0.97 (3H, d, *J* = 7.0 Hz), 0.75 (3H, t, *J* = 7.4 Hz). ^13^C NMR (50 MHz, CDCl_3_) δ 139.1, 129.0, 128.2, 126.9, 120.3, 64.0, 61.0, 54.9, 33.0, 27.1, 16.0, 11.2, −0.2.

(2*R*,3*S*)-3-(Dibenzylamino)-5-methyl-2-(trimethylsilyloxy) hexanonitrile (**2d**). Colorless oil. (5.5 g, 13.8 mmol, 88%). IR (neat, cm^−1^): 3061, 2955, 2228, 1255, 1106, 848 cm^−1. 1^H NMR (200 MHz, CDCl_3_) δ 7.34 (10H, m), 4.45 (1H, d, *J* = 5.8 Hz), 3.81 (2H, d, *J* = 13.8 Hz), 3.63 (2H, d, *J* = 13.8 Hz), 3.00 (1H, m), 1.75 (2H, m), 1.25 (1H, m), 0.90 (3H, d, *J* = 6.6 Hz), 0.70 (3H, d, *J* = 6.6 Hz). ^13^C NMR (50 MHz, CDCl_3_) δ 139.0, 128.6, 128.0, 126.8, 119.8, 63.2, 58.8, 54.6, 36.4, 25.0, 23.3, 22.0, 20.4.

(2*R*,3*S*)-3-(Dibenzylamino)-4-methyl-2-(trimethylsilyloxy) pentanonitrile (**2e**). Colorless oil. (3.9 g, 9.9 mmol, 99%). IR (neat, cm^−1^): 3062, 2957, 2218, 1250, 1085, 840 cm^−1. 1^H NMR (200 MHz, CDCl_3_) δ 7.32 (10H, m), 4.77 (1H, d, *J* = 4.4 Hz), 4.19 (2H, d, *J* = 13.2 Hz), 3.73 (2H, d, *J* = 13.2 Hz), 2.66 (1H, dd, *J* = 10.6, 5.8 Hz), 2.15 (1H, m), 1.02 (3H, d, *J* = 6.6 Hz), 0.97 (3H, d, *J* = 6.6 Hz). ^13^C NMR (50 MHz, CDCl_3_) δ 138.9, 129.2, 128.5, 127.4, 119.9, 64.6, 59.3, 54.6, 28.9, 22.4, 20.1, −0.2.

(2*S*,3*S*)-3-(Dibenzylamino)-2-(trimethylsilyloxy) butanonitrile (**3a**). Colorless oil. (2.7 g, 8.9 mmol, 80%). IR (neat, cm^−1^): 3061, 2959, 2212, 1253, 1092, 845 cm^−1. 1^H NMR (200 MHz, CDCl_3_) δ 7.43 (10H, m), 4.47 (1H, d, *J* = 8.8 Hz), 3.87 (2H, d, *J* = 13.6 Hz), 3.64 (2H, d, *J* = 13.6 Hz), 3.24 (1H, m), 1.24 (3H, d, *J* = 6.6 Hz). ^13^C NMR (50 MHz, CDCl_3_) δ 139.0, 128.8, 128.4, 127.2, 120.0, 65.2, 57.6, 55.1, 9.5, 0.08. ESI-MS, *m*/*z*: [M + H]^+^ 353; [M + Na]^+^ 375.

(2*S*,3*S*)-3-(Dibenzylamino)-4-phenyl-2-(trimethylsilyloxy) butanonitrile (**3b**). Colorless oil. (3.1 g, 8.7 mmol, 82%). IR (neat, cm^−1^): 3028, 2950, 2368, 1254, 1114, 850 cm^−1. 1^H NMR (200 MHz, CDCl_3_) δ 7.22 (15H, m), 4.46 (1H, d, *J* = 6.2 Hz), 3.72 (4H, br s), 3.38 (1H, dt, *J* = 8.0, 6.2 Hz), 2.99 (2H, m), 0.17 (9H, s). ^13^C NMR (50 MHz, CDCl_3_) δ 138.9, 138.6, 129.1, 128.5, 128.0, 127.9, 126.8, 126.0, 119.5, 63.0, 62.7, 54.7, 33.1, −0.4. ESI-MS, *m*/*z*: [M + H]^+^ 429; MS/MS, *m*/*z* (rel. int.): 311(100), 210(20). ESI-MS, *m*/*z*: [M + Na]^+^ 451.

(2*S*,3*S*)-3-(Dibenzylamino)-4-methyl-2-(trimethylsilyloxy) hexanonitrile (**3c**). Colorless oil. (3.8 g, 9.8 mmol, 74%). IR (neat, cm^−1^): 3060, 2958, 2232, 1245, 1102, 845 cm^−1. 1^H NMR (200 MHz, CDCl_3_) δ 7.33 (10H, m), 4.78 (1H, d, *J* = 4.8 Hz), 3.87 (2H, d, *J* = 13.6 Hz), 3.65 (2H, d, *J* = 13.6 Hz), 2.79 (1H, dd, *J* = 7.8, 4.8 Hz), 1.94 (2H, m), 1.17 (1H, m), 0.97 (3H, d, *J* = 7.0 Hz), 0.75 (3H, t, *J* = 7.4 Hz). ^13^C NMR (50 MHz, CDCl_3_) δ 139.1, 129.0, 128.2, 126.9, 120.3, 64.0, 61.0, 54.9, 33.0, 27.1, 16.0, 11.2, −0.2. ESI-MS, *m*/*z*: [M + H]^+^ 296; EM/EM *m*/*z* (rel. int.): 206(35), 181(95), 120(85), 91(100). EM-IES *m*/*z*: [M + H]^+^ 395, [M + Na]^+^ 417.

(2*S*,3*S*)-3-(Dibenzylamino)-5-methyl-2-(trimethylsilyloxy) hexanonitrile (**3d**). Colorless oil. (4.8 g, 12.6 mmol, 97%). IR (neat, cm^−1^): 3061, 2955, 2228, 1255, 1106, 848 cm^−1. 1^H NMR (200 MHz, CDCl_3_) δ 7.34 (10H, m), 4.45 (1H, d, *J* = 5.8 Hz), 3.81 (2H, d, *J* = 13.8 Hz), 3.63 (2H, d, *J* = 13.8 Hz), 3.00 (1H, m), 1.75 (2H, m), 1.25 (1H, m), 0.90 (3H, d, *J* = 6.6 Hz), 0.70 (3H, d, *J* = 6.6 Hz). ^13^C NMR (50 MHz, CDCl_3_) δ 139.0, 128.6, 128.0, 126.8, 119.8, 63.2, 58.8, 54.6, 36.4, 25.0, 23.3, 22.0, 20.4. ESI-MS, *m*/*z*: [M + H]^+^ 395, [M + Na]^+^ 417, [M-Me + 2H]^+^ 381, MS/MS, *m*/*z* (rel. int.): 290(100), 248(10), 213(12), 157(10).

(2*S*,3*S*)-3-(Dibenzylamino)-4-methyl-2-(trimethylsilyloxy) pentanonitrile (**3e**). Colorless oil. (6.0 g, 15.2 mmol, 96%). IR (neat, cm^−1^): 3062, 2957, 2218, 1250, 1085, 840 cm^−1. 1^H NMR (200 MHz, CDCl_3_) δ 7.32 (10H, m), 4.77 (1H, d, *J* = 4.4 Hz), 4.19 (2H, d, *J* = 13.2 Hz), 3.73 (2H, d, *J* = 13.2 Hz), 2.66 (1H, dd, *J* = 10.6, 5.8 Hz), 2.15 (1H, m), 1.02 (3H, d, *J* = 6.6 Hz), 0.97 (3H, d, *J* = 6.6 Hz). ^13^C NMR (50 MHz, CDCl_3_) δ 138.9, 129.2, 128.5, 127.4, 119.9, 64.6, 59.3, 54.6, 28.9, 22.4, 20.1, −0.2. ESI-MS, *m*/*z*: [M-Me + 2H]^+^ 367, MS/MS, *m*/*z* (rel. int.): 325(95), 275(100), 199(10).

**Scheme 3.**
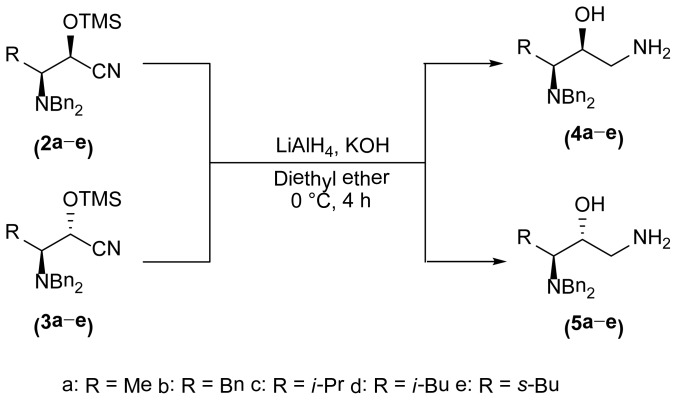
Synthesis of the aminoalcohols **4** and **5**.

A general procedure for the synthesis of the α-*N*,*N*-dibenzylaminoaminoalcohols **4** and **5** is described below (see Scheme 3).

LiAlH_4_ (2.0 equiv) was added to a solution of a trimetylsilyloxycianohydrine **3a** in anhydrous Et_2_O under argon atmosphere at 0 °C and the resulting mixture was stirred for 4 h at the same temperature. An aqueous solution of 5% potassium hydroxide (KOH) was added dropwise to the reaction mixture and a white solid was obtained. The resulting mixture was filtered and the solvent was removed under vacuum to obtain a crude oil.

(2*S*,3*S*)-1-Amino-3-(dibenzylamino)-2-butanol (**4a**). Colorless oil. (1.59 g, 5.1 mmol, 47%). IR (neat, cm^−1^): 3363, 3284, 3027, 2958, 1578, 1076 cm^−1. 1^H NMR (200 MHz, CDCl_3_) δ 7.25 (10H, m), 3.65 (2H, d, *J* = 13.6 Hz), 3.62 (1H, dd, *J* = 11.0, 5.8 Hz), 3.43 (1H, m), 3.31 (2H, d, *J* = 13.6 Hz), 2.76 (1H, dd, *J* = 11.0, 6.4 Hz), 2.55 (1H, q, *J* = 6.6 Hz), 1.10 (3H, d, *J* = 6.6 Hz). ^13^C NMR (50 MHz, CDCl_3_) δ 140.0, 129.1, 128.6, 127.2, 72.0, 66.0, 54.3, 44.7, 15.4.

(2*S*,3*S*)-1-Amino-3-(dibenzylamino)-4-phenyl-2-butanol (**4b**). Colorless oil. (3.4 g, 9.6 mmol, 94%). IR (neat, cm^−1^): 3367, 3298, 2924, 1603, 1252, 1109 cm^−1. 1^H NMR (200 MHz, CDCl_3_) δ 7.21 (15H, m), 3.93 (1H, dd, *J* = 13.4, 9.4 Hz), 3.70 (2H, d, *J* = 13.6 Hz), 3.57 (2H, d, *J* = 13.6 Hz), 3.43 (1H, dd, *J* = 15.0, 13.4, Hz), 2.82 (4H, m). ^13^C NMR (50 MHz, CDCl_3_) δ 141.1, 139.5, 129.1, 128.5, 128.2, 127.9, 126.6, 125.5, 72.0, 61.4, 54.4, 44.6, 32.5.

(2S,3*S*,4*S*)-1-Amino-3-(dibenzylamino)-4-methyl-2-hexanol (**4c**). Colorless oil. (2.3 g, 7 mmol, 72%). IR (neat, cm^−1^): 3345, 3289, 3060, 2955, 1601, 1376, 1061, 1027 cm^−1. 1^H NMR (200 MHz, CDCl_3_) δ 7.24 (10H, m), 3.75 (2H, d, *J* = 13.2 Hz), 3.73 (2H, m), 3.54 (2H, d, *J* = 13.2 Hz), 2.65 (1H, dd, *J* = 13.6, 7.0 Hz), 2.44 (1H, dd, *J* = 7.6, 4.6 Hz), 2.24 (1H, m), 1.90 (1H, m), 1.65 (1H, m), 1.08 (3H, d, *J* = 6.6 Hz), 0.95 (3H, t, *J* = 7.6 Hz). ^13^C NMR (50 MHz, CDCl_3_) δ 139.7, 128.8, 128.0, 126.8, 70.1, 62.5, 54.8, 44.6, 32.4, 29.8, 16.1, 12.2.

(2*S*,3*S*)-1-Amino-3-(dibenzylamino)-5-methyl-2-hexanol (**4d**). Colorless oil. (3.05 g, 6.5 mmol, 50%) IR (neat, cm^−1^): 3302, 3062, 2952, 1601, 1366, 1072 cm^−1. 1^H NMR (200 MHz, CDCl_3_) δ 7.30 (10H, m), 3.76 (1H, m), 3.62 (4H, br s), 3.38 (1H, dd, *J* = 13.2, 11.8 Hz), 2.66 (3H, m), 1.60 (1H, m), 0.91 (3H, d, *J* = 6.6 Hz), 0.73 (3H, d, *J* = 6.4 Hz). ^13^C NMR (50 MHz, CDCl_3_) δ 139.9, 128.7, 127.9, 126.6, 71.4, 56.8, 54.6, 44.9, 35.5, 25.2, 23.2, 22.7.

(2S,3S)-1-Amino-3-(dibenzylamino)-4-methyl-2-pentanol (**4e**). Colorless oil. (1.5 g, 4.8 mmol, 88%) IR (neat, cm^−1^): 3342, 3283, 3061, 2955, 1601, 1361, 1068 cm^−1. 1^H NMR (200 MHz, CDCl_3_) δ 7.24 (10H, m), 3.87 (2H, d, *J* = 13.2 Hz), 3.66 (2H, d, *J* = 13.2 Hz), 3.56 (1H, dd, *J* = 10.6, 4.8 Hz), 3.42 (1H, t, *J* = 10.6 Hz), 2.52 (1H, m), 2.06 (1H, m), 1.12 (3H, d, *J* = 6.6 Hz), 0.87 (1H, d, *J* = 7.0 Hz). ^13^C NMR (50 MHz, CDCl_3_) δ 139.0, 129.2, 128.2, 127.3, 69.1, 64.2, 54.2, 46.2, 26.4, 23.6, 19.6.

(2*R*,3*S*)-1-Amino-3-(dibenzylamino)-2-butanol (**5a**). Colorless oil. (3.3 g, 11.5 mmol, 76%). IR (neat, cm^−1^): 3363, 3284, 3027, 2958, 1578, 1076 cm^−1. 1^H NMR (200 MHz, CDCl_3_) δ 7.25 (10H, m), 3.65 (2H, d, *J* = 13.6 Hz), 3.62 (1H, dd, *J* = 11.0, 5.8 Hz), 3.43 (1H, m), 3.31 (2H, d, *J* = 13.6 Hz), 2.76 (1H, dd, *J* = 6.4, 11.0 Hz), 2.55 (1H, q, *J* = 6.6 Hz), 1.10 (3H, d, *J* = 6.6 Hz). ^13^C NMR (50 MHz, CDCl_3_) δ 140.3, 129.1, 128.5, 127.2, 73.5, 65.6, 54.7, 44.6, 12.1. ESI-MS, *m*/*z*: [M + H]^+^ 286; MS/MS, *m*/*z* (rel. Int.): 268(100), 199(5), 91(10).

(2*R*,3*S*)-1-Amino-3-(dibenzylamino)-4-phenyl-2-butanol (**5b**). Colorless oil. (3.4 g, 9.6 mmol, 94%). IR (neat, cm^−1^): 3365, 3299, 2926, 1600, 1251, 1110 cm^−1. 1^H NMR (200 MHz, CDCl_3_) δ 7.21 (15H, m), 3.93 (1H, dd, *J* = 13.4, 9.4 Hz), 3.70 (2H, d, *J* = 13.6 Hz), 3.57 (2H, d, *J* = 13.6 Hz), 3.43 (1H, dd, *J* = 15.0, 13.4 Hz), 2.82 (4H, m). ^13^C NMR (50 MHz, CDCl_3_) δ 141.1, 139.5, 129.1, 128.5, 128.2, 127.9, 126.6, 125.5, 72.0, 61.4, 54.4, 44.6, 32.5. ESI-MS, *m*/*z*: [M + H]^+^ 361; MS/MS, *m*/*z* (rel. int.): 344(98), 300(20), 210(100).

(2*R*,3*S*)-1-Amino-3-(dibenzylamino)-4-methyl-2-hexanol (**5c**). Colorless oil. (2.4 g, 7.35 mmol, 79%). IR (neat, cm^−1^): 3345, 3289, 3060, 2955, 1601, 1376, 1061, 1027 cm^−1. 1^H NMR (200 MHz, CDCl_3_) δ 7.24 (10H, m), 3.75 (2H, d, *J* = 13.2 Hz), 3.73 (2H, m), 3.54 (2H, d, *J* = 13.2 Hz), 2.65 (1H, dd, *J* = 13.6, 7.0 Hz), 2.44 (1H, dd, *J* = 7.6, 4.6 Hz), 2.24 (1H, m), 1.90 (1H, m), 1.65 (1H, m), 1.08 (3H, d, *J* = 6.6 Hz), 0.95 (3H, t, *J* = 7.6 Hz). ^13^C NMR (50 MHz, CDCl_3_) δ 139.7, 128.8, 128.0, 126.8, 70.1, 62.5, 54.8, 44.6, 32.4, 29.8, 16.1, 12.2. ESI-MS, *m*/*z*: [M + H]^+^ 327; MS/MS *m*/*z* (rel. int.): 309(100), 292(10), 198(35), 91(5).

(2*R*,3*S*)-1-Amino-3-(dibenzylamino)-5-methyl-2-hexanol (**5d**). Colorless oil. (3.05 g, 6.5 mmol, 50%). IR (neat, cm^−1^): 3300, 3061, 2952, 1601, 1366, 1072 cm^−1. 1^H NMR (200 MHz, CDCl_3_) δ 7.30 (10H, m), 3.76 (1H, m), 3.62 (4H, br s), 3.38 (1H, dd, *J* = 13.2, 11.8 Hz), 2.66 (3H, m), 1.60 (1H, m), 0.91 (3H, d, *J* = 6.6 Hz), 0.73 (3H, d, *J* = 6.4 Hz). ^13^C NMR (50 MHz, CDCl_3_) δ 139.9, 128.7, 127.9, 126.6, 71.4, 56.8, 54.6, 44.9, 35.5, 25.2, 23.2, 22.7. ESI-MS, *m*/*z*: [M + H]^+^ 327; MS/MS, *m*/*z* (rel.int.): 310(100), 233(45), 198(30).

(2*R*,3*S*)-1-Amino-3-(dibenzylamino)-4-methyl-2-pentanol (**5e**). Colorless oil. (2.8 g, 8.7 mmol, 60%). IR (neat, cm^−1^): 3342, 3283, 3061, 2955, 1601, 1361, 1068 cm^−1. 1^H NMR (200 MHz, CDCl_3_) δ 7.24 (10H, m), 3.87 (2H, d, *J* = 13.2 Hz), 3.66 (2H, d, *J* = 13.2 Hz), 3.56 (1H, dd, *J* = 10.6, 4.8 Hz), 3.42 (1H, t, *J* = 10.6 Hz), 2.52 (1H, m), 2.06 (1H, m), 1.12 (3H, d, *J* = 6.6 Hz), 0.87 (1H, d, *J* = 7.0 Hz). ^13^C NMR (50 MHz, CDCl_3_) δ 139.5, 128.9, 128.0, 126.8, 70.5, 64.0, 55.1, 44.7, 26.3, 23.6, 20.1. ESI-MS, *m*/*z*: [M + H]^+^ 313; MS/MS, *m*/*z* (rel. int.): 296(100), 278(5), 199(10), 91(7).

**Scheme 4.**
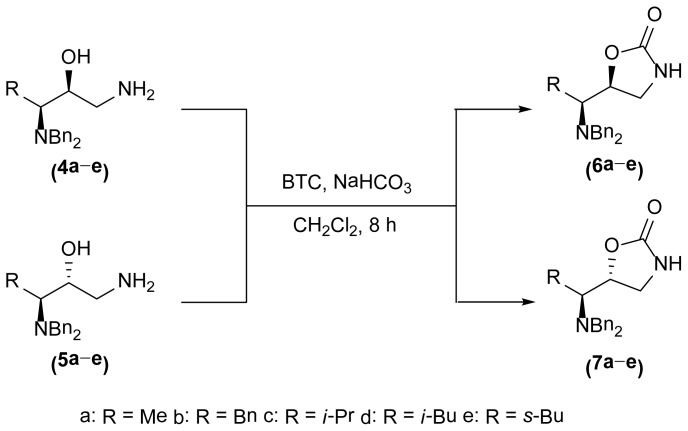
Synthesis of oxazolidinone analogues **6** and **7**.

A general procedure for the synthesis of 1,3-oxazolidin-2-ones **6** and **7** is described below (see Scheme 4).

α-*N*,*N*-dibencilaminoaminoalcohol **5a** (3.0 g, 10.4 mmol) was dissolved in anhydrous DCM (100 mL). A solution of triphosgene (1 equiv) in anhydrous DCM was added dropwise to the aminoalcohol **5a** solution at 0 °C. The reaction mixture was stirred for 8 h, followed by addition of saturated sodium bicarbonate (NaHCO_3_) and the resulting mixture was stirred for an additional 30 min. The mixture was extracted with DCM (3 × 20 mL). The organic layers were combined, and it was dried with anhydrous Na_2_SO_4_, filtered and the solvent was removed under vacuum. The residue was purified by column chromatography over silica gel to afford the desired product.

(*S*)-5-((*S*)-1-(Dibenzylamino)ethyl)oxazolidin-2-one (**6a**). Light brown solid. (2.3 g, 6.6 mmol, 99%). IR (neat, cm^−1^, KBr): 3277, 2961, 1752, 1237, 1077 cm^−1. 1^H NMR (200 MHz, CDCl_3_) δ 7.27 (10H, m), 5.49 (1H, br s), 4.86 (1H, q, *J* = 8.4 Hz), 3.73 (2H, d, *J* = 13.6 Hz), 3.66 (1H, t, *J* = 8.4 Hz), 3.48 (2H, d, *J* = 13.6 Hz), 3.26 (1H, t, *J* = 8.4 Hz), 2.66 (1H, dd, *J* = 9.0, 1.6 Hz), 2.03 (1H, m), 1.44 (2H, m), 1.14 (3H, d, *J* = 7.0 Hz), 0.93 (3H, t, *J* = 7.2 Hz). ^13^C NMR (50 MHz, CDCl_3_) δ 159.9, 139.2, 129.0, 128.4, 127.3, 75.4, 63.6, 53.7, 44.7, 30.7, 30.0, 14.6, 11.4.

(*S*)-5-((*S*)-1-(Dibenzylamino)-2-phenylethyl)oxazolidin-2-one (**6b**). Light brown solid. (2.5 g, 6.4 mmol, 89%). IR (neat, cm^−1^, KBr): 3283, 3026, 2931, 1754, 1368, 1240 cm^−1. 1^H NMR (200 MHz, CDCl_3_) δ 7.21 (15H, m), 5.91 (1H, br s), 4.80 (1H, m), 3.62 (4H, br s), 3.52 (1H, t, *J* = 8.8 Hz), 3.08 (1H, t, *J* = 8.8 Hz), 3.06 (3H, br s). ^13^C NMR (50 MHz, CDCl_3_) δ 159.6, 139.5, 138.8, 129.3, 128.4, 128.1, 128.0, 127.9, 126.8, 126.0, 76.8, 62.4, 54.4, 44.8, 32.4.

(5*S*)-5-((1*S*)-1-(Dibenzylamino)-2-methylbutyl)oxazolidin-2-one (**6c**). Colorless oil. (2.11 g, 5.9 mmol, 84%). IR (neat, cm^−1^, KBr): 3277, 2961, 1752, 1237, 1077 cm^−1. 1^H NMR (200 MHz, CDCl_3_) δ 7.27 (10H, m), 5.49 (1H, br s), 4.86 (1H, q, *J* = 8.4 Hz), 3.73 (2H, d, *J* = 13.6 Hz), 3.66 (1H, t, *J* = 8.4 Hz), 3.48 (2H, d, *J* = 13.6 Hz), 3.26 (1H, t, *J* = 8.4 Hz), 2.66 (1H, dd, *J* = 9.0, 1.6 Hz), 2.03 (1H, m), 1.44 (2H, m), 1.14 (3H, d, *J* = 7.0 Hz), 0.93 (3H, t, *J* = 7.2 Hz). ^13^C NMR (50 MHz, CDCl_3_) δ 159.9, 139.2, 129.0, 128.4, 127.3, 75.4, 63.6, 54.7, 45.7, 31.7, 30.0, 15.6, 12.4.

(*S*)-5-((*S*)-1-(Dibenzylamino)-3-methylbutyl)-1,3-oxazolidin-2-one (**6d**). Colorless oil (3.5 g, 9.8 mmol, 90%). IR (neat, cm^−1^, KBr): 3277, 3028, 1753, 1240, 1080 cm^−1. 1^H NMR (200 MHz, CDCl_3_) δ 7.27 (10H, m), 6.09 (1H, br s), 4.75 (1H, m), 3.72 (2H, d, *J* = 13.8 Hz), 3.58 (1H, t, *J* = 8.4 Hz), 3.55 (2H, d, *J* = 13.8 Hz), 3.12 (1H, t, *J* = 8.4 Hz), 2.67 (1H, dd, *J* = 7.0, 5.6 Hz), 1.96 (1H, heptet, *J* = 6.6 Hz), 1.73 (6H, q, *J* = 7.0 Hz), 1.27 (1H, m), 0.92 (3H, d, *J* = 6.6 Hz), 0.73 (3H, d, *J* = 6.2 Hz).^13^C NMR (50 MHz, CDCl_3_) δ 160.3, 140.1, 129.4, 128.4, 127.1, 78.9, 57.0, 55.0, 43.9, 35.6, 24.5, 23.4, 22.4.

(*S*)-5-((*S*)-1-(Dibenzylamino)-2-methylpropyl)-1,3-oxazolidin-2-one (**6e**). Light brown solid. (1.5 g, 4.4 mmol, 50%). IR (neat, cm^−1^, KBr): 3277, 3028, 1753, 1240, 1080 cm^−1. 1^H NMR (200 MHz, CDCl_3_) δ 7.27 (10H, m), 6.09 (1H, br s), 4.75 (1H, m), 3.72 (2H, d, *J* = 13.8 Hz), 3.58 (1H, t, *J* = 8.4 Hz), 3.55 (2H, d, *J* = 13.8 Hz), 3.12 (1H, t, *J* = 8.4 Hz), 2.67 (1H, dd, *J* = 7.0, 5.6 Hz), 1.96 (1H, heptet, *J* = 6.6 Hz), 1.73 (6H, q, *J* = 7.0 Hz), 1.27 (1H, m), 0.92 (3H, d, *J* = 6.6 Hz), 0.73 (3H, d, *J* = 6.2 Hz). ^13^C NMR (50 MHz, CDCl_3_) δ 160.1, 139.9, 129.4, 128.3, 127.0, 73.4, 63.6, 56.1, 44.0, 28.2, 21.4, 19.1.

(*R*)-5-((*S*)-1-(Dibenzylaminoethyl)-1,3-oxazolidin-2-one (**7a**). Light brown solid. (2.7 g, 8.6 mmol, 85%). ${\left[\alpha \right]}_{D}^{20}=+21.2$ (*c* 1.00, CHCl_3_). P*_f_* = 95 °C. *R_f_* = 0.48 (ethyl acetate/petroleum ether: 1/1). IR (neat, cm^−1^, KBr): 3254, 2826, 1748, 1239 cm^−1. 1^H NMR (200 MHz, CDCl_3_) δ 7.25 (10H, m), 5.73 (1H, br s), 4.83 (1H, q, *J* = 8.6 Hz), 3.67 (2H, d, *J* = 13.4 Hz), 3.62 (1H, t, *J* = 8.6 Hz), 3.55 (2H, d, *J* = 13.4 Hz), 3.23 (1H, t, *J* = 8.6 Hz), 2.58 (1H, dd, *J* = 8.6, 3.2 Hz), 3.33 (1H, m), 1.11 (6H, t, *J* = 7.4 Hz). ^13^C NMR (50 MHz, CDCl_3_) δ 159.7, 138.8, 128.7, 128.1, 127.0, 78.3, 56.5, 54.4, 44.7, 8.6. ESI-MS, *m*/*z*: [M + H]^+^ 311; MS/MS *m*/*z* (rel. int.): 250(50), 219(100), 181(93), 91(60). HPLC: 0.8 mL/min; 70:30 MeOH/H_2_O; *R_t_*: 5.8 min.

(*R*)-5-((*S*)-1-(Dibenzylamino)-2-phenylethyl)oxazolidin-2-one (**7b**). Light brown solid. (2.5 g, 6.4 mmol, 89%). ${\left[\alpha \right]}_{D}^{20}=+8.0$ (*c* 1.00, CHCl_3_). P*_f_* = 102 °C. *R_f_* = 0.50 (ethyl acetate/petroleum ether: 1/1). IR (neat, cm^−1^, KBr): 3283, 3026, 2931, 1754, 1368, 1240 cm^−1. 1^H NMR (200 MHz, CDCl_3_) δ 7.21 (15H, m), 5.91 (1H, br s), 4.80 (1H, m), 3.62 (4H, br s), 3.52 (1H, t, *J* = 8.8 Hz), 3.08 (1H, t, *J* = 8.8 Hz), 3.06 (3H, br s). ^13^C NMR (50 MHz, CDCl_3_) δ 159.6, 139.5, 138.8, 129.3, 128.4, 128.1, 128.0, 127.9, 126.8, 126.0, 76.8, 62.4, 54.4, 44.8, 32.4. ESI-MS, *m*/*z*: [M + H]^+^ 387; MS/MS *m*/*z* (rel. int.): 326(75), 295(100), 181(40). HPLC: 0.8 mL/min; 70:30 MeOH/H_2_O; *R_t_*: 6.3 min.

(5*R*)-5-((1*S*)-1-(Dibenzylamino)-2-methylbutyl)oxazolidin-2-one (**7c**). Light brown solid. (2.3 g, 6.6 mmol, 99%). ${\left[\alpha \right]}_{D}^{20}=+11.5$ (*c* 1.00, CHCl_3_). P*_f_* = 42 °C. *R_f_* = 0.62 (ethyl acetate/petroleum ether: 1/1). IR (neat, cm^−1^, KBr): 3277, 2961, 1752, 1237, 1077 cm^−1. 1^H NMR (200 MHz, CDCl_3_) δ 7.27 (10H, m), 5.49 (1H, br s), 4.86 (1H, q, *J* = 8.4 Hz), 3.73 (2H, d, *J* = 13.6 Hz), 3.66 (1H, t, *J* = 8.4 Hz), 3.48 (2H, d, *J* = 13.6 Hz), 3.26 (1H, t, *J* = 8.4 Hz), 2.66 (1H, dd, *J* = 9.0, 1.6 Hz), 2.03 (1H, m), 1.44 (2H, m), 1.14 (3H, d, *J* = 7.0 Hz), 0.93 (3H, t, *J* = 7.2 Hz). ^13^C NMR (50 MHz, CDCl_3_) δ 159.9 (C), 139.2, 129.0, 128.4, 127.3, 75.4, 63.6, 54.7, 45.7, 31.7, 30.0, 15.6, 12.4. ESI-MS, *m*/*z*: [M + H]^+^ 353; MS/MS, *m*/*z* (rel. int.): 283(100), 202(45). HPLC: 0.8 mL/min; 70:30 MeOH/H_2_O; *R_t_*: 8.4 min.

(*R*)-5-((*S*)-1-(Dibenzylamino)-3-methylbutyl)-oxazolidin-2-one (**7d**). Light brown solid. (1.5 g, 4.4 mmol, 50%). ${\left[\alpha \right]}_{D}^{20}=-30.9$ (*c* 1.00, CHCl_3_). P*_f_* = 43 °C. *R_f_* = 0.52 (ethyl acetate/petroleum ether: 1/1). IR (neat, cm^−1^, KBr): 3277, 3028, 1753, 1240, 1080 cm^−1. 1^H NMR (200 MHz, CDCl_3_) δ 7.27 (10H, m), 6.09 (1H, br s), 4.75 (1H, m), 3.72 (2H, d, *J* = 13.8 Hz), 3.58 (1H, t, *J* = 8.4 Hz), 3.55 (2H, d, *J* = 13.8 Hz), 3.12 (1H, t, *J* = 8.4 Hz), 2.67 (1H, dd, *J* = 7.0, 5.6 Hz), 1.96 (1H, heptet, *J* = 6.6 Hz), 1.73 (6H, q, *J* = 7.0 Hz), 1.27 (1H, m), 0.92 (3H, d, *J* = 6.6 Hz), 0.73 (3H, d, *J* = 6.2 Hz). ^13^C NMR (50 MHz, CDCl_3_) δ 159.9, 139.2, 128.6, 128.0, 126.9, 76.8, 58.2, 54.4, 44.9, 35.1, 24.7, 23.3, 22.4. ESI-MS, *m*/*z*: [M + H]^+^ 353; MS/MS *m*/*z* (rel. int.): 261(100), 202(75), 181(80). HPLC: 0.8 mL/min; 70:30 MeOH/H_2_O; *R_t_*: 6.8 min.

(*R*)-5-((*S*)-1-(Dibenzylamino)-2-methylpropyl)oxazolidin-2-one (**7e**). Light brown solid. (1.5 g, 4.0 mmol, 85%). ${\left[\alpha \right]}_{D}^{20}=-9.0$ (*c* 1.00, CHCl_3_). P*_f_* = 45 °C. *R_f_* = 0.55 (ethyl acetate/petroleum ether: 1/1). IR (neat, cm^−1^, KBr): 3284, 2958, 1752, 1239, 1078 cm^−1. 1^H NMR (200 MHz, CDCl_3_) δ 7.25 (10H, m), 5.73 (1H, br s), 4.83 (1H, q, *J* = 8.6 Hz), 3.67 (2H, d, *J* = 13.4 Hz), 3.62 (1H, t, *J* = 8.6 Hz), 3.55 (2H, d, *J* = 13.4 Hz), 3.23 (1H, t, *J* = 8.6 Hz), 2.58 (1H, dd, *J* = 8.6, 3.2 Hz), 3.33 (1H, m), 1.11 (6H, t, *J* = 7.4 Hz). ^13^C NMR (50 MHz, CDCl_3_) δ 159.7, 138.8, 128.7, 128.1, 127.0, 75.4, 64.1, 54.6, 45.5, 25.1, 23.3, 19.0. ESI-MS, *m*/*z*: [M + H]^+^ 339; MS/MS, *m*/*z* (rel.int.): 283(100), 247(40), 188(45). HPLC: 0.8 mL/min; 70:30 MeOH/H_2_O; *R_t_*: 5.8 min.

## 4. Conclusions

A series of syn- and anti-diasteromeric 1,3-oxazolidin-2-ones have been synthesized in good yields, and were evaluated against MRSA clinical isolates. The oxazolidinone **7a** has shown antimicrobial activity against the tested isolates. In addition, preliminary toxicity studies using brine shrimp have demonstrated that the synthesized compounds present low toxicity and the most active compound **7a** could be a potential antimicrobial agent with low toxicity against the tested MRSA isolates.

## Figures and Tables

**Table 1.** Synthesis of 1,3-oxazolidin-2-ones **6** and **7**.

Entry	Compound	% Yield (6/7)
1	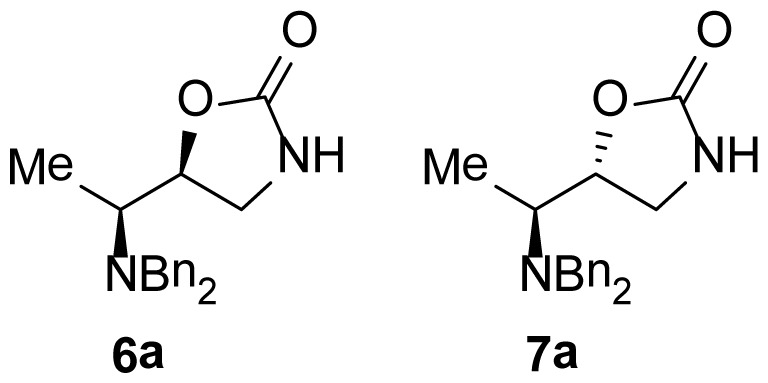	47/92
2	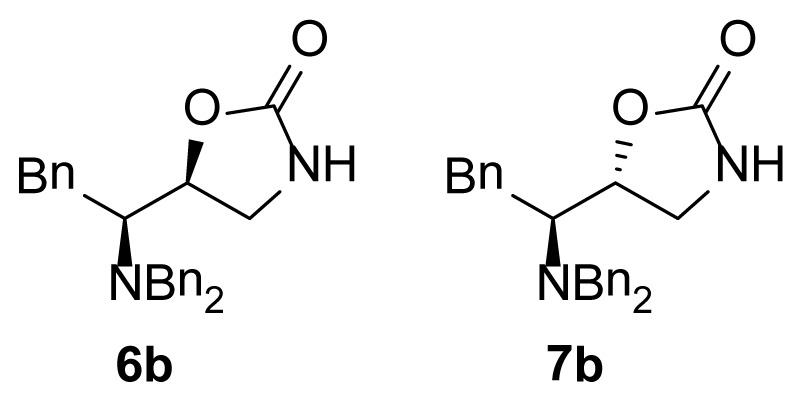	89/94
3	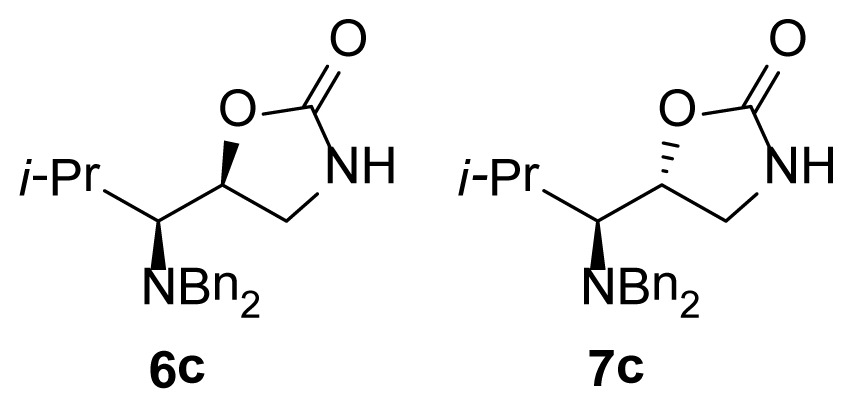	92/92
4	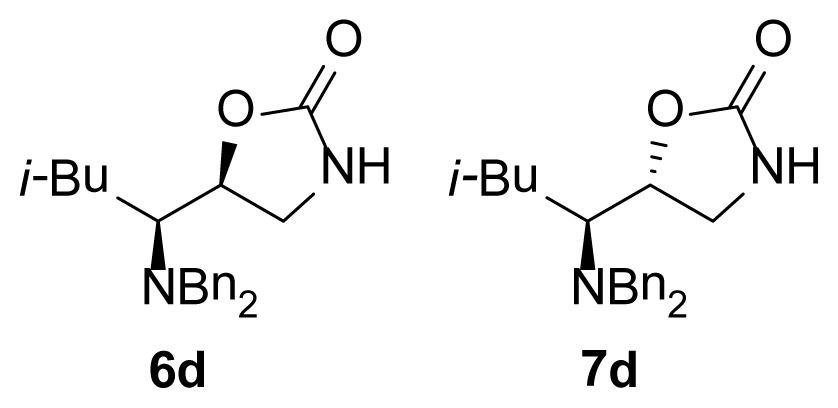	73/90
5	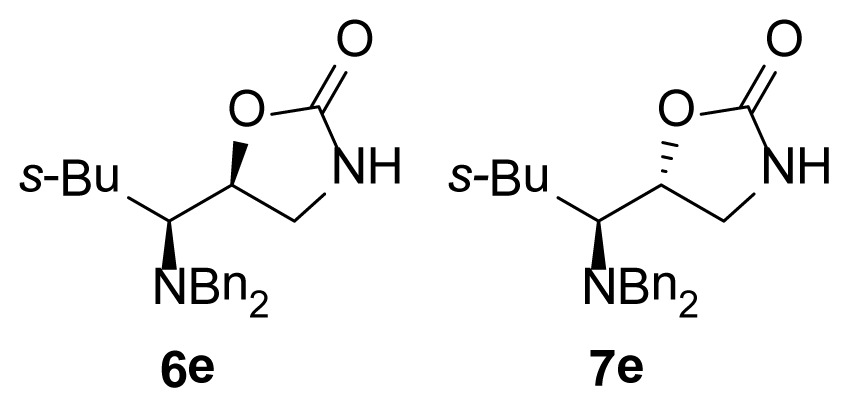	84/99

**Table 2.** Inhibitory activity (mm) of standard antibiotics * and **7a** oxazolidinone analogue on growth of methicillin resistant *S. aureus* (MRSA) clinical isolates (means ± SD, *n* = 3).

Isolates	C	N	I	C/AC	V	L	Oxazolidinone 7a		
						
	10 μg	10 μg	10 μg	30 μg	30 μg	10 μg	20 μg	10 μg	6.6 μg
SMC-27	R	R	S	S	S	S	12.5 (±0.09)	8.9 (±0.05)	4.5 (±0.05)
SMC-47	S	R	S	R	S	S	12.6 (±0.08)	9.1 (±0.05)	4.6 (±0.07)
SMC-48	R	R	S	R	S	S	12.5 (±0.05)	8.8 (±0.06)	4.4 (±0.05)
SMC-57	S	R	S	R	S	S	12.7 (±0.05)	9.2 (±0.04)	4.8 (±0.05)
SMC-71	S	S	R	S	S	S	0	0	0
SMC-79	R	S	S	R	S	S	0	0	0
SMC-87	R	R	S	R	S	S	12.6 (±0.05)	8.9 (±0.05)	4.4 (±0.04)
SMC-97	R	S	R	R	S	S	12.9 (±0.06)	9.3 (±0.06)	4.5 (±0.04)
SMC-124	S	0	S	R	S	S	12.8 (±0.06)	9.1 (±0.07)	4.6 (±0.05)
SMC-141	R	S	R	R	S	S	12.9 (±0.05)	8.9 (±0.05)	4.5 (±0.05)
SMC-159	R	S	S	R	S	S	12.8 (±0.06)	8.8 (±0.06)	4.6 (±0.04)
SMC-162	S	R	S	R	S	S	12.5 (±0.07)	9.2 (±0.05)	4.5 (±0.04)

* N = Norfloxacin; I = Imipenem; C = Ceftazidime; C/CA = Ceftazidime/Clavulanic acid; V = Vancomycin; L = Linezolid.
